# Pulmonary mucosa‐associated lymphoid tissue lymphoma mimicking lung cancer

**DOI:** 10.1002/ccr3.2276

**Published:** 2019-06-19

**Authors:** Shinichi Miyazaki, Kensuke Hachiya, Yoshiharu Nara, Takuya Ikeda

**Affiliations:** ^1^ Department of Respiratory Medicine Yokkaichi Municipal Hospital Yokkaichi‐shi Japan; ^2^ Department of Hematology Yokkaichi Municipal Hospital Yokkaichi‐shi Japan; ^3^ Department of Pathology Yokkaichi Municipal Hospital Yokkaichi‐shi Japan

**Keywords:** lung cancer, MALT lymphoma

## Abstract

Pulmonary mucosa‐associated lymphoid tissue (MALT) lymphoma is the most frequent subset of primary pulmonary lymphoma. MALT lymphoma may manifest as a solid mass, mimicking lung cancer.

## 1 CASE DESCRIPTION

**Figure 1:**
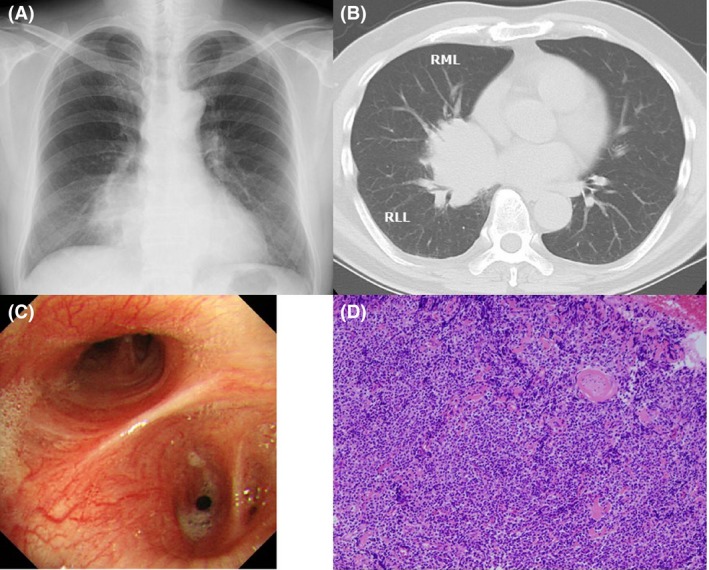
A, Chest radiograph on presentation. B, Computed tomography scan of the chest on presentation (RML; right middle lobe, RLL; right lower lobe). C, Bronchoscopic examination showing no endobronchial abnormalities in right middle and inferior lobe bronchi. D, Specimen obtained by transbronchial lung biopsy (hematoxylin and eosin stain; original magnification ×100)

An asymptomatic 64‐year‐old man who is an ex‐smoker was referred to our hospital because of an abnormal chest radiograph during a routine medical check‐up. Physical examination was unremarkable; however, a chest radiograph revealed a lower lung mass (Figure 1A), and a computed tomography (CT) scan confirmed a lung mass in the right hilum with stenosis of right middle and inferior lobe bronchi (Figure 1B). During fiberoptic bronchoscopy, no endobronchial abnormalities were noted (Figure 1C). Transbronchial biopsy specimens revealed diffuse infiltration of the lung parenchyma by small and medium‐sized lymphoid cells (Figure 1D). These cells were CD20‐positive and bcl2‐positive, but did not co‐express CD5, CD10, bcl6, or cyclin D1. Because the systemic examination revealed no other lesions, primary mucosa‐associated lymphoid tissue (MALT) lymphoma of the lung was diagnosed. The patient underwent six cycles of R‐CHOP (rituximab, cyclophosphamide, doxorubicin, vincristine, and prednisone), resulting in complete remission of the tumor.

Primary pulmonary lymphoma represents only 0.5% of all primary lung neoplasms, with pulmonary MALT lymphoma being the most frequent subset. MALT lymphoma manifests as diverse lung abnormality patterns on CT scans; the most common findings are nodules, masses, and patchy consolidations with air bronchogram.^1^ MALT lymphoma may also manifest as a solid mass, mimicking lung cancer.

## CONFLICT OF INTEREST

None declared.

## AUTHOR CONTRIBUTIONS

SM: cared for the patient and wrote the manuscript. KH: helped prepare the manuscript. YN: made the pathological diagnosis and contributed in capturing microscopic images. TI: contributed to critical reading of the manuscript.

## INFORMED CONSENT

Informed consent was obtained from the patient to conduct this study.
